# Comparison of invisalign mandibular advancement and twin-block on upper airway and hyoid bone position improvements for skeletal class II children: a retrospective study

**DOI:** 10.1186/s12903-023-03295-2

**Published:** 2023-09-13

**Authors:** Zheng Yue, Zian Yi, Xinyi Liu, Mengting Chen, Shuhui Yin, Qianqian Liu, Xuefeng Chen, Jiangtian Hu

**Affiliations:** 1 Department of Orthodontics, Kunming Medical University School and Hospital of Stomatology, Kunming, 650031 Yunnan China; 2 Department of Orthodontics, Lianbang Institute of Stomatological Technology and Hospital of Stomatology, Xi’an, 710032 Shaanxi China; 3 State Key Laboratory of Military Stomatology and National Clinical Research Center for Oral Diseases and Shaanxi Key Laboratory of Stomatology, Department of Orthodontics, School of Stomatology, The Fourth Military Medical University, Xi’an, 710032 Shaanxi China; 4 Xuefeng Dental Care, Huaian, 223000 Jiangsu China

**Keywords:** Skeletal class II malocclusion, Invisalign mandibular advancement, Hyoid bone, Twin-block, Upper airway

## Abstract

**Background:**

This study is to evaluate and compare the improvement of upper airway morphology and hyoid bone position in children with Class II mandibular retrusion treated with Invisalign mandibular advancement (MA) and Twin-Block (TB) appliances, utilizing cone beam computed tomography (CBCT).

**Methods:**

32 children aged between 8 and 11.5 years old were included in this study, with an average age of 10.2 years old. These children were divided into two groups, MA and TB, with 16 children in each group. Changes in upper airway morphology and hyoid bone position before and after treatment were analyzed using CBCT.

**Results:**

(1) Changes in upper airway before and after treatment: the oropharynx volume (Or-V), the oropharynx minimum cross-sectional area (Or-mCSA), the hypopharynx volume (Hy-V), and the hypopharynx minimum cross-sectional area (Hy-mCSA) in both the MA and TB groups increased after treatment, and the differences were statistically significant (*P* < 0.05) compared to pre-treatment status. (2) Changes in hyoid bone position before and after treatment: The distances between H point and third cervical vertebra (H-C3), H point and pogonion (H-RGN), H point and mandibular plane (H-MP), H point and Frankfort horizontal plane (H-FH), H and S point (H-S), and H point and palatal plane (H-PP) in both the MA and TB groups increased after treatment, and the differences were statistically significant (*P* < 0.05).

**Conclusion:**

Both MA and TB appliances effectively improved the structural narrowness of the upper airway and reduced respiratory resistance, thus improving breath quality. However, MA showed more effectiveness in improving the narrowest part of the hypopharynx compared to TB. Both appliances also promoted anterior downward movement of the hyoid bone, which opens the upper airway of the oropharynx and hypopharynx and helps the upper airway morphology return to normal range.

**Supplementary Information:**

The online version contains supplementary material available at 10.1186/s12903-023-03295-2.

## Background

Skeletal Class II malocclusion is a type of dentofacial deformity characterized by a deficiency of the mandible, or prognathism of the maxilla, or the concurrent presence of both features [1]. The abnormal growth and position of the maxilla and mandible will lead to abnormalities of both craniofacial morphology and upper airway morphology. The abnormality of the upper airway morphology will in turn affect the growth and development of the craniofacial tissue [2, 3]. Therefore, it is crucial to include the upper airway morphology into clinical analysis and treatment strategy during early correction of skeletal Class II.

The hyoid bone is a small, unique bone in the human body that is located freely between the chin and neck, while not connected to any other bones. There is a complex relationship among growth and development abnormalities of the maxilla and mandible, position changes of the hyoid bone, and morphology alterations of the upper airway. Currently, there are many studies focusing on this area [4–7]. Changes in the sagittal and vertical dimensions and positions of the mandible will affect the position of the hyoid bone. Retrusion of the mandible can cause posterior displacement of the hyoid bone, while clockwise rotation of the mandible can lead to inferior-posterior displacement of the hyoid bone [4].

It has been proved by previous studies that upper airway narrowing is the most crucial factor in obstructive sleep apnea (OSA), which has long-term adverse effect on more than 2% of the children [8, 9]. Therefore, a detailed assessment of the upper airway and hyoid bone is essential for routine orthodontic planning and treatment outcome assessment of this type of malocclusion.

Twin-block (TB) is an extensively used functional appliance. The appliance is mainly applied to skeletal retrognathia growing patients. It was proved to be an effective treatment in improving the morphology of the upper airway and the position of the hyoid bone in children and adolescents with mandibular retrusion [10]. Invisalign mandibular advancement (MA) is a novel functional invisible appliance primarily used for skeletal Class II growing patients with mandibular retrusion. Although the working principle of MA is similar to that of Twin-block appliance, it is not exactly the same [11].

Most previous studies assessed alterations in the upper airway through cephalometric radiography. However, this method restricts the accuracy of upper airway measurements as it only depicts anteroposterior changes within the sagittal plane, lacking a full-scale view of the upper airway [12]. Hence, it is imperative to establish a three-dimensional (3D) evaluation of the upper airway in growing patients undergoing MA and TB treatment.

In this study, we used Dolphin Imaging 11.9 software and cone beam computed tomography (CBCT) data to reconstruct a 3D model of the upper airway to evaluate the volume and the minimum cross-sectional area (mCSA) of each segment of the upper airway. Additionally, we utilized lateral cephalometric radiographs to evaluate the changes in the position of the hyoid bone.

The aim of this study is to investigate the changes in upper airway and hyoid bone position before and after MA and TB treatment in patients with skeletal Class II malocclusion [13, 14]. Additionally, we compared the effectiveness of these two treatment methods to provide guidance for clinical practitioners. This study elucidated the relationship between orthodontic correction of dental malocclusions and the upper airway morphology as well as the hyoid bone position. Currently, there is limited research on the impact of MA on the upper airway and hyoid bone to theoretically support clinical treatment.

## Materials and methods

### Subject

Thirty-two children (15 boys and 17 girls, with age range 8-11.5 years, and with an average age of 10.2 ± 0.84 years) were selected from the Orthodontic Department of the Affiliated Stomatological Hospital of Kunming Medical University between February 2019 and February 2022.

The following detailed inclusion criteria were utilized for all patients [15, 16]:

1. Mixed dentition or early permanent dentition, crowding < 4 mm, the canines and molars have a distal relationship, deep overbite: I°-III°, deep overjet: I°-III°.
2. Skeletal Class II (ANB angle > 4°), normal maxilla (78.8° ≤ SNA angle ≤ 85.8°), mandibular retrognathia (SNB ≤ 77.6°).
3. Growth patterns were hypodivergent or normal (20° ≤ FH-MP angle ≤ 32°).
4. Cervical vertebral maturation stage between CS2 and CS3 [17].
5. Normal adenoid size (nasopharynx posterior airway width ≥ 10mm), and no obvious space-occupying lesions in the upper airway.

Patients information in each group was summarized as follows: (1) The MA group comprised 16 patients (7 boys and 9 girls, mean age 10.02 ± 0.99 years) diagnosed with skeletal Class II malocclusion, mandibular retrognathia. These patients had completed MA treatment and were selected for the MA group. (2) The TB group included 16 patients (8 boys and 8 girls, mean age 10.38 ± 0.68 years) diagnosed with skeletal Class II malocclusion, mandibular retrognathia. These patients had completed TB treatment and were well-matched with the patients in the MA group in terms of age, gender, and growth pattern. They were selected for the TB group.

No significant difference was noted between the 2 groups regarding sex and age (Table 1).

**Table 1:** Characteristics of the sample

Group	MA group	TB group	*p* value
Sex (n)			0.723*
Female	9	8	
Male	7	8	
Age, y (mean ± SD)	10.02 ± 0.99	10.38 ± 0.68	0.130^†^

SD, Standard deviation

*Pearson chi-square test; ^†^matched t-test

### Methods

**Fig. 1:**
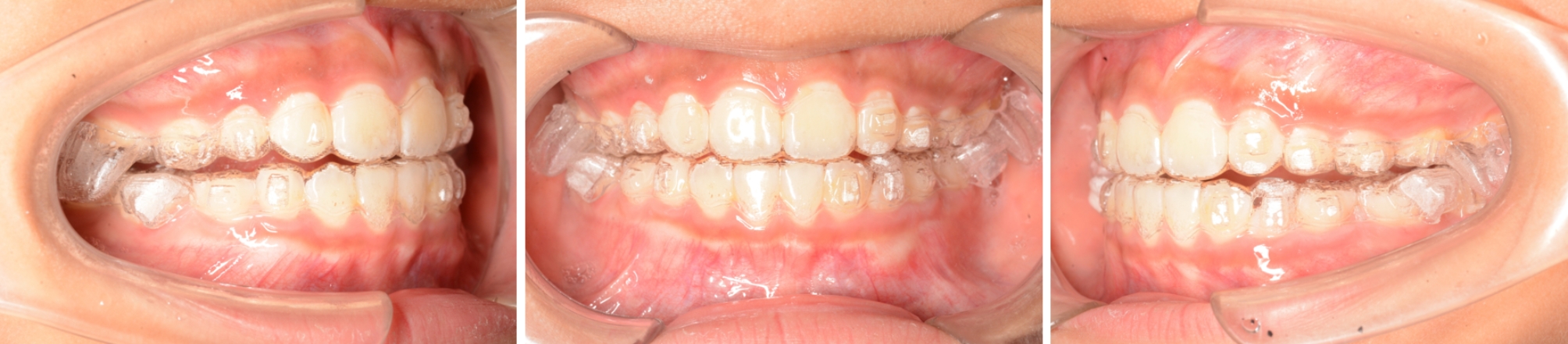
Patient of MA group with MA appliance in the mouth

MA group: The data of the patients were uploaded to the Invisalign design website for the treatment plan and aligners manufacture. The patients were instructed to wear the aligners for at least 22 hours a day, which can be removed for eating and brushing teeth. They were also instructed to replace the aligners every week. Follow-up appointments were scheduled every 4–6 weeks (Fig. 1).

**Fig. 2:**
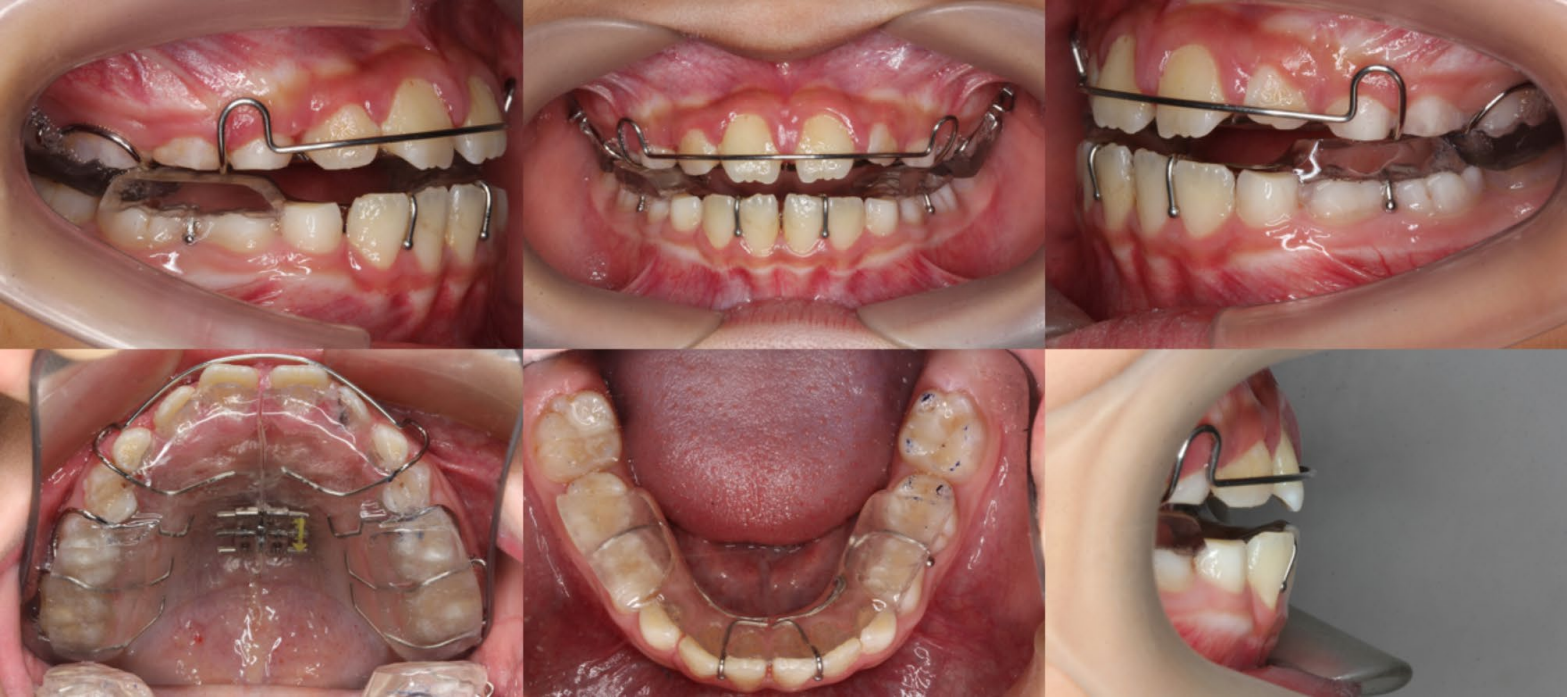
Patient of TB group with TB appliance in the mouth

TB group: The mandible was advanced at least 5–7 mm in the sagittal direction and opened 3–5 mm between the upper and lower canines. The bite records and working models were sent to the dental lab for TB fabrication. Follow-up appointments were scheduled every 4–6 weeks for monitoring, and the occlusal splints are adjusted by 1–2 mm at each visit (Fig. 2).

The treatment duration for the MA group was 11.45 ± 1.1 months, while for the TB group, it was 12.11 ± 1.3 months. At the end of the treatment, all patients exhibited Class I molar relationship, and had normal overjet and overbite of anterior tooth. Two cases from each group and presented their data including photos and 3D CBCT images of the upper airway volume in the supplementary materials (Additional file 1, sFig 1–4).

### Image acquisition

All image data were acquired by the same CBCT scanner (NewTom VGi, QR Verona, Italy), with all the patients maintain an upright sitting position, a natural head posture, a resting position of the tongue and a maximum intercuspation without swallowing. In both groups, the pre-assessment was conducted within two weeks prior to treatment initiation, while the post-assessment was performed immediately after treatment completion. CBCT datasets are exported in DICOM (Digital Imaging and Communications in Medicine) format.

### Image analysis

**Fig. 3:**
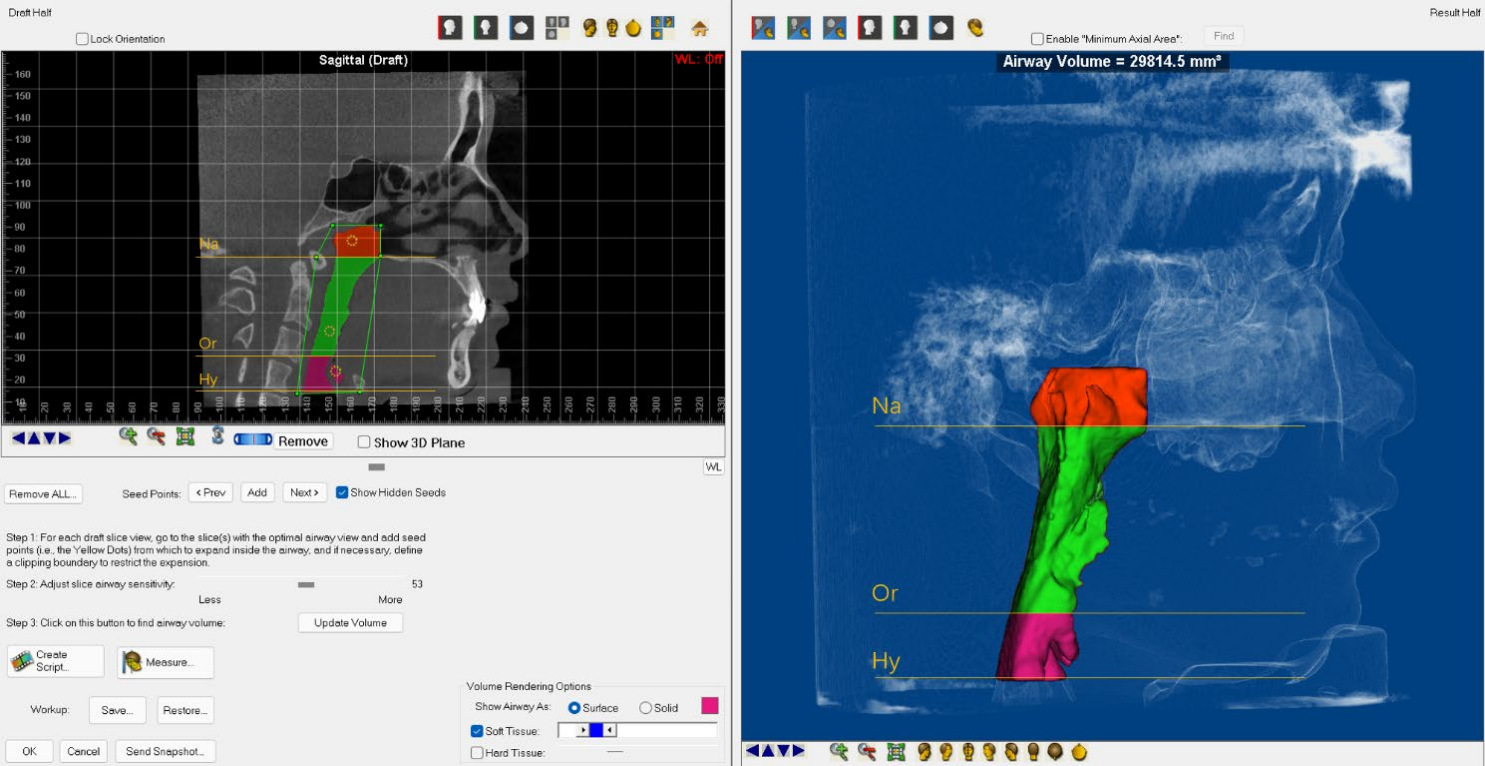
A 3D model of the upper airway was reconstructed and divided into three regions using two planes perpendicular to the sagittal plane. The planes were defined using two landmarks: the posterior nasal spine (PNS) and the superior border of the epiglottis (SE). The nasopharynx (Na), oropharynx (Or) and hypopharynx (Hy) were reconstructed separately to facilitate the analysis of upper airway morphology

All DICOM data were imported into Dolphin Imaging 11.9 software (Chatsworth, California, USA) to establish a three-dimensional upper airway model. The images were first reoriented to have Frankfort horizontal plane (FH) parallel to the ground. The Sinus/Airway Measurement Function was then used to reconstruct a three-dimensional model of the upper airway, which was divided into three parts: nasopharynx (Na, pharyngeal apex plane to posterior nasal spine plane), oropharynx (Or, posterior nasal spine plane to epiglottic apex plane) and hypopharynx (Hy, epiglottic apical plane to epiglottic basal plane). After the airway was reconstructed, the volume (V) and the minimum cross-sectional area of each segment were automatically calculated by Dolphin 3D (Fig. 3) [18, 19].

**Fig. 4:**
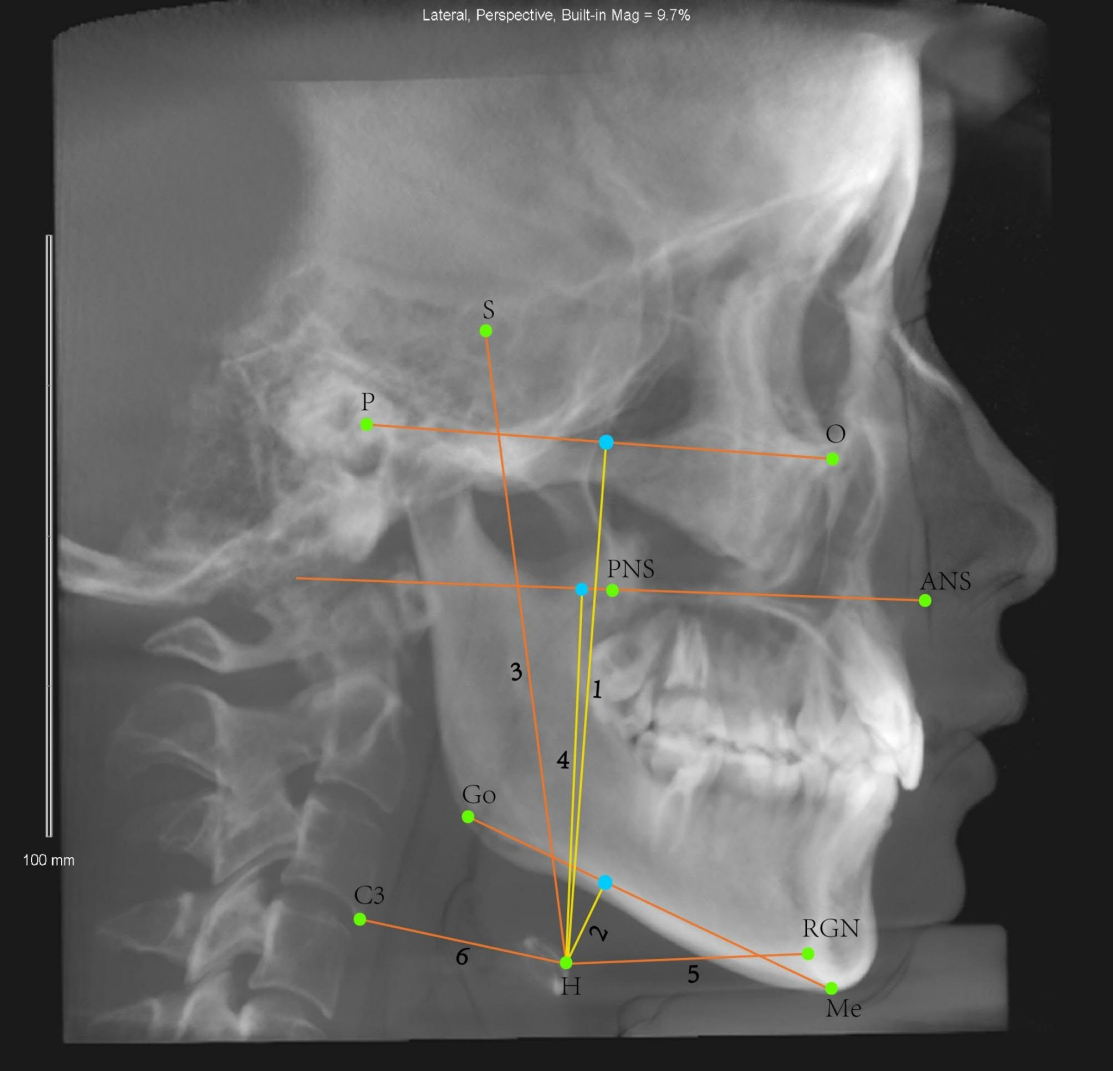
Hyoid bone linear measurements: **1** H-FH (mm), distance from H to Frankfort horizontal plane; **2** H-MP (mm), distance from H to mandibular plane; **3** H-S (mm), distance between H and S; **4** H-PP (mm), distance from H to the palatal plane; **5** H-RGN (mm), distance between H and RGN; **6** H-C3 (mm), distance between H and C3

The study utilized lateral cephalograms acquired through the Build X-ray Function to evaluate changes in the position of the hyoid bone. The definitions of landmarks and measurement variables are detailed in Tables 2, 3 and Fig. 4. The measurements were performed by the same researcher and were repeated three times to ensure accuracy, with the average measurement value used in our analysis.

**Table 2:** Hyoid bone position measurement landmarks

Abbreviation	Definition
S (sella)	Centre of the sella turcica; the centre of the pituitary fossa of the sphenoid bone
P (porion)	The most superior point of the external auditory meatus
O (orbitale)	The most inferior point of the orbit
PNS (posterior nasal spine)	The most posterior point of the hard palate
ANS (anterior nasal spine)	The apex of the anterior nasal spine
RGN (retrognation)	The most posterior point of the symphysis
Me (menton)	The most inferior point on the bony chin
C3 (third cervical vertebra)	The most anterior and inferior point of the third cervical vertebra
H (hyoid)	The most anterior and superior point of the hyoid bone
Go (gonion)	The most posteroinferior point on the angle of the mandible

**Table 3:** Dolphin Imaging Measurement Variables

Abbreviation	Full name	Definition
V (mm^3^)	Volume	The volume of each region of the upper airway (nasopharynx, oropharynx and hypopharynx)
mCSA (mm^2^)	Minimum cross-sectional area	The minimum cross-sectional area of each region of the upper airway
H-FH (mm)	Hyoid bone to Frankfort horizontal plane	The perpendicular distance from H to Frankfort horizontal plane
H-MP (mm)	Hyoid bone to mandibular plane	Perpendicular distance from H to mandibular plane
H-S (mm)	Hyoid bone to sella	Distance between H and sella
H-PP (mm)	Hyoid bone to palatal plane	The perpendicular distance from H to the palatal plane
H-RGN (mm)	Hyoid bone to retrognation	Distance between H and RGN
H-C3 (mm)	Hyoid bone to the third cervical vertebra	Distance between H and C3

## Results

### Changes in the upper airway before and after treatment with MA and TB

After treatment, both the MA and TB groups showed a statistically significant increase in the oropharynx volume (Or-V), oropharynx minimum cross-sectional area (Or-mCSA), hypopharynx volume (Hy-V), and hypopharynx minimum cross-sectional area (Hy-mCSA) (*P* < 0.05), while the changes in the nasopharynx volume (Na-V) and minimum cross-sectional area (Na-mCSA) were not statistically significant (*P* > 0.05) (Tables 4 and 5). These results suggest that MA and TB treatment led to a significant enlargement of the oropharynx and hypopharynx airway segments with increased volume, however, no significant effect on the nasopharynx segment.

**Table 4:** Morphological changes of upper airway before and after treatment in MA group

Variables	MA group (n = 16)						*t*	*P*
	Before		After		Δ (After - Before)			
	Mean	SD	Mean	SD	Mean	SD		
Na-V (mm^3^)	5547.16	2036.33	6159.76	2720.44	612.6	1420.48	1.725	0.105
Na-mCSA (mm^2^)	300.79	93.66	303.19	98.03	2.4	40.16	0.239	0.814
Or-V (mm^3^)	10925.48	3034.04	12617.37	3605.87	1691.89	1574.67	4.298	0.001**
Or-mCSA (mm^2^)	227.03	59.95	277.23	129.07	50.19	89.81	2.236	0.041*
Hy-V (mm^3^)	3470.14	1008.6	4304.57	1464.94	834.42	762.79	0.001**	0.000**
Hy-mCSA (mm^2^)	230.4	62.01	273.7	70.44	43.3	39.02	4.439	0.000**

* *p* < 0.05 ** *p* < 0.01

**Table 5:** Comparison of upper airway morphology before and after treatment in TB group

Variables	TB group (n = 16)						*t*	*P*
	Before		After		Δ (After - Before)			
	Mean	SD	Mean	SD	Mean	SD		
Na-V (mm^3^)	4344.84	2851.07	4709.44	3832.21	364.60	1019.93	-1.430	0.173
Na-mCSA (mm^2^)	208.05	110.29	214.84	97.75	6.79	17.00	-1.597	0.131
Or-V (mm^3^)	6903.13	1686.72	9097.69	746.34	2194.56	1437.15	-6.108	0.000**
Or-mCSA (mm^2^)	122.29	33.42	176.19	77.93	53.90	56.41	-3.822	0.002**
Hy-V (mm^3^)	1973.96	345.18	2681.71	389.41	707.75	613.99	-4.611	0.000**
Hy-mCSA (mm^2^)	150.65	29.98	163.94	42.92	13.29	15.68	-3.391	0.004**

* *p* < 0.05 ** *p* < 0.01

### Changes in hyoid bone position before and after treatment with MA and TB

After treatment, the distances between the hyoid bone and the third cervical vertebra (H-C3), retrognation (H-RGN), the mandibular plane (H-MP), Frankfort horizontal plane (H-FH), centre of the sella turcica (H-S), and palatal plane (H-PP) all increased significantly in both the MA and TB groups (*P* < 0.05) (Tables 6 and 7). This suggests that the hyoid bone position in patients treated with both MA and TB moved forward and downward.

**Table 6:** Comparison of hyoid bone position before and after treatment in MA group

Variables	MA group (n = 16)						*t*	*p*
	Before		After		Δ (After - Before)			
	Mean	SD	Mean	SD	Mean	SD		
H-C3 (mm)	35.94	5.23	37.69	4.05	1.75	2.77	2.528	0.023*
H-RGN (mm)	39.00	5.14	42.69	8.20	3.69	4.33	3.405	0.004**
H-MP (mm)	19.31	5.38	26.00	7.11	6.69	4.91	5.449	0.000**
H-FH (mm)	82.63	8.62	93.19	9.83	10.56	2.92	14.467	0.000**
H-S (mm)	104.44	10.30	110.38	8.57	5.94	6.29	3.778	0.002**
H-PP (mm)	61.38	5.74	67.94	9.29	6.56	7.04	3.727	0.002**

* *p* < 0.05 ** *p* < 0.01

**Table 7:** Comparison of hyoid bone position before and after treatment in TB group

Variables	TB group (n = 16)						*t*	*p*
	Before		After		Δ (After - Before)			
	Mean	SD	Mean	SD	Mean	SD		
H-C3 (mm)	30.44	3.44	33.13	3.98	2.69	2.91	3.688	0.002**
H-RGN (mm)	36.50	5.34	39.50	3.10	3.00	3.48	3.445	0.004**
H-MP (mm)	15.56	3.95	19.44	1.03	3.88	3.70	4.185	0.001**
H-FH (mm)	82.06	4.36	92.94	11.08	10.88	8.29	5.250	0.000**
H-S (mm)	98.94	6.64	105.38	4.32	6.44	5.07	5.076	0.000**
H-PP (mm)	56.94	8.16	61.94	6.51	5.00	5.90	3.390	0.004**

* *p* < 0.05 ** *p* < 0.01

### Comparison of upper airway changes between MA group and TB group before and after treatment

There were no statistically significant differences in the changes of the nasopharynx volume (Na-V), nasopharynx minimum cross-sectional area (Na-mCSA), oropharynx volume (Or-V), oropharynx minimum cross-sectional area (Or-mCSA) and hypopharynx volume (Hy-V) between MA group and TB group before and after treatment (*P* < 0.05), but there was a significant difference in the changes of the hypopharynx minimum cross-sectional area (Hy-mCSA) between the two groups before and after treatment (*P* < 0.05) (Table 8), suggesting that MA is more effective than TB in dilating the upper airway hypopharynx obstruction site.

**Table 8:** Intergroup comparison of the effects of MA and TB groups on upper airway morphology

Variables	Δ (After - Before)				*t*	*p*
	MA group		TB group			
	Mean	SD	Mean	SD		
Na-V (mm^3^)	612.60	1420.48	364.60	1019.93	-0.567	0.575
Na-mCSA (mm^2^)	2.40	40.16	6.79	17.00	0.402	0.692
Or-V (mm^3^)	1691.89	1574.67	2194.56	1437.15	0.943	0.353
Or-mCSA (mm^2^)	50.19	89.81	53.90	56.41	0.140	0.890
Hy-V (mm^3^)	834.42	762.79	707.75	613.99	-0.517	0.609
Hy-mCSA (mm^2^)	43.30	39.02	13.29	15.68	-2.855	0.010**

* *p* < 0.05 ** *p* < 0.01

### Comparison of hyoid bone position changes between MA group and TB group before and after treatment

There were no statistically significant differences (*P* > 0.05) in the changes of the distance between the hyoid bone and various reference points before and after treatment, including the third cervical vertebra (H-C3), retrognation (H-RGN), mandibular plane (H-MP), Frankfort horizontal plane (H-FH), centre of the sella turcica (H-S), and palatal plane (H-PP) between the MA and TB groups (Table 9). This suggests that the two appliances had no significant difference in their effect on the position of the hyoid bone.

**Table 9:** Comparison of the effects of MA and TB groups on hyoid bone position

Variables	Δ (After - Before)				*t*	*p*
	MA group		TB group			
	Mean	SD	Mean	SD		
H-C3 (mm)	1.75	2.77	2.69	2.91	0.933	0.358
H-RGN (mm)	3.69	4.33	3.00	3.48	-0.495	0.624
H-MP (mm)	6.69	4.91	3.88	3.70	-1.830	0.077
H-FH (mm)	10.56	2.92	10.88	8.29	0.142	0.888
H-S (mm)	5.94	6.29	6.44	5.07	0.248	0.806
H-PP (mm)	6.56	7.04	5.00	5.90	-0.680	0.502

* *p* < 0.05 ** *p* < 0.01

## Discussion

### Changes in upper airway morphology

The volume and minimum cross-sectional area of the upper airway in the oropharynx and hypopharynx significantly increased (*P* < 0.05) after treatment with MA and TB in patients with skeletal Class II mandibular retrusion. This suggests that the upper airway expanded significantly in the oropharynx and hypopharynx after the mandible was guided forward. These results are consistent with previous studies [20]. The possible reasons are as follows: First, the forward movement of the mandible in the spatial position causes the suprahyoid and extrinsic tongue muscles attached to the mandible to extend forward, pulling and driving the tongue body, hyoid bone, and soft tissues of the front wall of the oropharynx and hypopharynx to move forward. This movement expands the upper airway of the larynx, greatly reducing the compression of the uvula. Consequently, the palatopharyngeal segment behind the uvula becomes more unobstructed [21]. Second, the forward movement of the mandible also expands the oral cavity space, increasing the tongue’s range of motion. As the mandible moves forward, the tongue repositions forward, and the tongue base moves forward to tense the palate and pharyngeal walls. This increased tension reduces the likelihood of collapse of the upper airway of the oropharynx during inhalation in sleep, significantly improving the quality of ventilation [22]. The changes of nasopharynx volume (Na-V) and the nasopharynx minimum cross-sectional area (Na-mCSA) were not statistically significant (*P* > 0.05). There may be two potential factors could limit the upper airway dilatation in the nasopharynx segment. First, the oropharynx and hypopharynx are not surrounded by hard bone tissue, and therefore are more susceptible to changes caused by the forward movement of the mandible. In contrast, the nasopharynx segment is surrounded by hard bony structures, making it less susceptible to environmental changes [23]. Second, the nasopharynx segment of the upper airway is more closely related to the maxilla than the mandible and therefore is less affected by changes in mandible position [24].

### Changes in hyoid bone position

In this study, all variables related to the hyoid bone exhibited significant increases (*P* < 0.05). These findings were consistent with the previous study by DA COSTA [25]. Anteroinferior displacement of the hyoid bone after correction may have two possible causes. Firstly, the anterior displacement of the mandible leads to the anterior displacement of the suprahyoid, extrahyoid, and hyoglossus muscles attached to it. These muscles pull the hyoid bone and tongue body forward in the sagittal direction, resulting in the expansion of oropharynx tissues around the upper airway [26, 27]. Secondly, both MA and TB promote the anterior displacement of the mandible, but the anterior mandible also rotates clockwise as the hyoid bone gradually moves downward with age, causing the vertical downward movement of the hyoid bone [28]. Treatment with either appliance displaced the hyoid bone anteriorly and inferiorly, disrupting the balance and coordination of the muscle chain of the stomatognathic system, shifting the position of the tongue body, increasing the volume of the upper airway, and causing morphological changes [19, 29].

### Comparison of the effects of MA and TB on the upper airway

After treatment, there was a significantly greater increase in the hypopharynx minimum cross-sectional area (Hy-mCSA) in the MA group compared to the TB group (*P* < 0.05), indicating that MA more significantly improved the narrowest area of the hypopharynx airway than TB. Studies have shown that several specific orthodontic treatments for patients with skeletal Class II malocclusion, such as extraction orthodontic treatment or orthodontic treatment using implant anchorage to intrude the dentition, vertical control by moving the occlusal fulcrum forward and reducing the occlusal height of posterior teeth, can cause the mandible and occlusal plane to rotate counterclockwise to varying degrees, thereby increasing the upper airway volume, especially in the oropharynx and hypopharynx segments [30–32]. MA can intrude the anterior teeth while advancing the mandible forward and establish occlusion in the desired position without extruding the posterior teeth [33]. However, TB can not intrude the anterior teeth during the treatment and usually requires multiple grinding occlusal splints to guide the posterior teeth to extrude to establish occlusion [34]. Thus, MA is superior to TB in terms of controlling vertical mandibular position and producing less clockwise rotation after mandibular advancement. This may be the critical factor in significantly improving the minimum cross-sectional area of the hypopharynx after treatment in the MA group compared to the TB group.

### Comparison of the effects of MA and TB on the hyoid bone

The post-treatment changes in all variables related to the hyoid bone showed significant increases in both the MA and TB groups (*P* < 0.05). However, there was no statistical difference in the comparison of changes between the two groups before and after treatment (*P* > 0.05), indicating that both appliances can effectively promote anterior displacement of the hyoid bone and that their therapeutic effects are similar. Although there are some differences between the two treatment methods in terms of their effects on leading the mandible forward, such as control of the mandibular plane angle. The differences in the improvement of the mandible between the two groups are not sufficient to cause significant differences in the position of the hyoid bone, since the hyoid bone is primarily connected to the mandible and tongue through muscles and ligaments and is affected by muscle compensation.

### Advantages and disadvantages of MA compared to TB

Under the guidance of MA and TB, the minimum cross-sectional area and volume of the oropharynx and hypopharynx airways behind the mandible are significantly increased, and the hyoid bone is also moved forward and downward to avoid narrowing or even collapse and obstruction of the upper airway, effectively preventing the occurrence of OSA. In addition to its aesthetic and comfortable advantages, MA has the following advantages compared with TB: 1. Three-dimensional control can be achieved simultaneously. For patients with malocclusion and mandibular retrognathism with deep overbite, MA can be used to create spaces for tooth alignment through maxillary expansion and molar distalization, shortening the course of orthodontic treatment [35]. 2. It has a wider range of indications. MA can be used to correct Class II high-angle malocclusion, which is a contraindication for traditional functional appliances such as TB [36]. 3. MA has a better improvement effect on the minimum cross-sectional area of the upper airway in the hypopharynx segment than TB, so MA has an advantage in relieving hypopharynx airway obstruction. In clinical practice, if the narrowest part of the upper airway occurs mainly in the hypopharynx segment, MA may be considered as better option due to its efficacy. However, there are some disadvantages and limitations to MA such as its price [37].

### Limitations

This study utilized upright CBCT data to investigate the impact of orthodontic treatment on airway improvement, aiming to demonstrate its potential in reducing the risk of OSA. It should be noted that in supine position, the narrowing of the upper airway tends to worsen, especially in patients with OSA. The influence of body position is acknowledged as a limitation of this study, and future research is recommended to incorporate supine imaging data for a more comprehensive evaluation.

## Conclusions

Both MA and TB appliances showed success in improving the structural narrowness of the upper airway and reduced respiratory resistance. However, MA showed more effectiveness in improving the narrowest part of the hypopharynx compared to TB. Both appliances also promoted anterior downward movement of the hyoid bone, which opens the upper airway of the oropharynx and hypopharynx and helps the upper airway morphology return to normal range.

### Electronic supplementary material

Below is the link to the electronic supplementary material.

**Additional file 1:** The additional file 1 includes 4 figures depicting pre- and post-treatment data, reflecting two cases each from the MA group and the TB group: **sFig 1**, Case #1 of MA group. A-C, pre-treatment photos; D-F, post-treatment photos; G, pre-treatment 3D rendering of upper airway volume (Na-V = 2099.9 mm^3^, Or-V = 11799.5 mm^3^, Hy-V = 4059.9 mm^3^); H, post-treatment 3D rendering of upper airway volume (Na-V = 4532.5 mm^3^, Or-V = 13030.1 mm^3^, Hy-V = 5425.0 mm^3^); **sFig 2**, Case #2 of MA group. A-C, pre-treatment photos; D-F, post-treatment photos; G, pre-treatment 3D rendering of upper airway volume (Na-V = 8051.5 mm3, Or-V = 11968.1 mm3, Hy-V = 3609.2 mm3); H, post-treatment 3D rendering of upper airway volume (Na-V = 8706.8 mm3, Or-V = 13124.8 mm3, Hy-V = 4507.7 mm3); **sFig 3**, Case #1 of TB group. A-C, pre-treatment photos; D-F, post-treatment photos; G, pre-treatment 3D rendering of upper airway volume (Na-V = 1067.5 mm3, Or-V = 6957.5 mm3, Hy-V = 4452.4 mm3); H, post-treatment 3D rendering of upper airway volume (Na-V = 2257.2 mm3, Or-V = 8114.4 mm3, Hy-V = 5513.1 mm3); **sFig 4**, Case #2 of TB group. A-C, pre-treatment photos; D-F, post-treatment photos; G, pre-treatment 3D rendering of upper airway volume (Na-V = 5512.2 mm3, Or-V = 13793.8 mm3, Hy-V = 4186.8 mm3); H, post-treatment 3D rendering of upper airway volume(Na-V = 5002.4 mm3, Or-V = 21058.7 mm3, Hy-V = 5914.9 mm3).

## Data Availability

The datasets used and/or analysed during the current study are available from the corresponding author on reasonable request.

## List of abbreviations

3D: Three Dimensional
CBCT: Cone Beam Computer Tomography
CS: Cervical Vertebral Maturation Stages
DICOM: Digital Imaging and Communications in Medicine
MA: Mandibular Advancement
OSA: Obstructive Sleep Apnea
TB: Twin-Block

## References

[1] Lv W, Nie Q, Gu Y (2021). Three-dimensional analysis of mandibular characteristics in patients with skeletal class II malocclusion and chin deviation. Am J Orthod Dentofac Orthopedics: Official Publication Am Association Orthodontists Its Constituent Soc Am Board Orthod.

[2] Veasey SC, Rosen IM (2019). Obstructive sleep apnea in adults. N Engl J Med.

[3] Batool-Anwar S, Goodwin JL, Kushida CA, Walsh JA, Simon RD, Nichols DA, Quan SF (2016). Impact of continuous positive airway pressure (CPAP) on quality of life in patients with obstructive sleep apnea (OSA). J Sleep Res.

[4] Liu Y, Chen W, Wei Y, Zhang G, Zhang X, Sharhan HM, Cao B (2022). The effect of orthodontic vertical control on the changes in the upper airway size and tongue and hyoid position in adult patients with hyperdivergent skeletal class II. BMC Oral Health.

[5] Hartfield PJ, Janczy J, Sharma A, Newsome HA, Sparapani RA, Rhee JS, Woodson BT, Garcia GJM (2022). Anatomical determinants of upper airway collapsibility in obstructive sleep apnea: a systematic review and meta-analysis. Sleep Med Rev.

[6] Samaha CJ, Tannous HJ, Salman D, Ghafari JG, Amatoury J (2022). Role of surgical hyoid bone repositioning in modifying upper airway collapsibility. Front Physiol.

[7] Jo JH, Park JW, Jang JH, Chung JW (2022). Hyoid bone position as an indicator of severe obstructive sleep apnea. BMC Pulm Med.

[8] Leiter JC (1996). Upper airway shape: is it important in the pathogenesis of obstructive sleep apnea?. Am J Respir Crit Care Med.

[9] Wang R, Mihaicuta S, Tiotiu A, Corlateanu A, Ioan IC, Bikov A (2022). Asthma and obstructive sleep apnoea in adults and children - an up-to-date review. Sleep Med Rev.

[10] Baka ZM, Fidanboy M (2021). Pharyngeal airway, hyoid bone, and soft palate changes after class II treatment with twin-block and Forsus appliances during the postpeak growth period. Am J Orthod Dentofacial Orthop.

[11] Caruso S, Nota A, Caruso S, Severino M, Gatto R, Meuli S, Mattei A, Tecco S (2021). Mandibular advancement with clear aligners in the treatment of skeletal class II. A retrospective controlled study. Eur J Paediatr Dent.

[12] Aboudara C, Nielsen I, Huang JC, Maki K, Miller AJ, Hatcher D (2009). Comparison of airway space with conventional lateral headfilms and 3-dimensional reconstruction from cone-beam computed tomography. Am J Orthod Dentofacial Orthop.

[13] Garcia-Usó M, Lima TF, Trindade IEK, Pimenta LAF, Trindade-Suedam IK (2021). Three-dimensional tomographic assessment of the upper airway using 2 different imaging software programs: a comparison study. Am J Orthod Dentofacial Orthop.

[14] Guijarro-Martínez R, Swennen GRJ (2013). Three-dimensional cone beam computed tomography definition of the anatomical subregions of the upper airway: a validation study. Int J Oral Maxillofac Surg.

[15] Han M, Wang RY, Liu H, Zhu XJ, Wei FL, Lv T, Wang NN, Hu LH, Li GJ, Liu DX (2013). Association between mandibular posterior alveolar morphology and growth pattern in a chinese population with normal occlusion. J Zhejiang Univ Sci B.

[16] Idris G, Hajeer MY, Al-Jundi A (2012). Acceptance and discomfort in growing patients during treatment with two functional appliances: a randomised controlled trial. Eur J Paediatr Dent.

[17] Baccetti T, Franchi L, McNamara JA (2002). An improved version of the cervical vertebral maturation (CVM) method for the assessment of mandibular growth. Angle Orthod.

[18] de Water VR, Saridin JK, Bouw F, Murawska MM, Koudstaal MJ (2014). Measuring upper airway volume: accuracy and reliability of Dolphin 3D software compared to manual segmentation in craniosynostosis patients. J Oral Maxillofac Surg.

[19] Arens R, Marcus CL. Pathophysiology of upper airway obstruction: a developmental perspective. Sleep 2004, 27(5). 10.1093/sleep/27.5.997 15453561

[20] Shete CS, Bhad WA (2017). Three-dimensional upper airway changes with mandibular advancement device in patients with obstructive sleep apnea. Am J Orthod Dentofacial Orthop.

[21] Tsuiki S, Lowe AA, Almeida FR, Kawahata N, Fleetham JA (2004). Effects of mandibular advancement on airway curvature and obstructive sleep apnoea severity. Eur Respir J.

[22] Jugé L, Knapman FL, Humburg P, Burke PGR, Lowth AB, Brown E, Butler JE, Eckert DJ, Ngiam J, Sutherland K et al. The relationship between mandibular advancement, tongue movement, and treatment outcome in obstructive sleep apnea. Sleep 2022, 45(6). 10.1093/sleep/zsac044 35218653

[23] Teresi LM, Lufkin RB, Vinuela F, Dietrich RB, Wilson GH, Bentson JR, Hanafee WN (1987). MR imaging of the nasopharynx and floor of the middle cranial fossa. Part I. normal anatomy. Radiology.

[24] Sayinsu K, Isik F, Arun T (2006). Sagittal airway dimensions following maxillary protraction: a pilot study. Eur J Orthod.

[25] da Costa ED, Roque-Torres GD, Brasil DM, Bóscolo FN, de Almeida SM, Ambrosano GMB (2017). Correlation between the position of hyoid bone and subregions of the pharyngeal airway space in lateral cephalometry and cone beam computed tomography. Angle Orthod.

[26] Auvenshine RC, Pettit NJ. The hyoid bone: an overview. Cranio 2020, 38(1). 10.1080/08869634.2018.1487501 30286692

[27] Bilal R. Position of the Hyoid Bone in Anteroposterior Skeletal Patterns. *J Healthc Eng* 2021, 2021:7130457. 10.1155/2021/7130457 PMC8424248 34512939

[28] Wu Y, Yu Q, Xia Y, Wang B, Chen S, Gu K, Zhang B, Zhu M (2023). Does mandibular advancement with clear aligners have the same skeletal and dentoalveolar effects as traditional functional appliances?. BMC Oral Health.

[29] Amatoury J, Kairaitis K, Wheatley JR, Bilston LE, Amis TC (2015). Peripharyngeal tissue deformation, stress distributions, and hyoid bone movement in response to mandibular advancement. J Appl Physiol (1985).

[30] Ning R, Guo J, Martin D (2022). Effect of premolar extraction on upper airway volume and hyoid position in hyperdivergent adults with different mandibular length. Am J Orthod Dentofac Orthopedics: Official Publication Am Association Orthodontists Its Constituent Soc Am Board Orthod.

[31] Shi X, Chen H, Lobbezoo F, Berkhout E, de Lange J, Guo J, Aarab G (2021). Effects of miniscrew-assisted orthodontic treatment with premolar extractions on upper airway dimensions in adult patients with class II high-angle malocclusion. Am J Orthod Dentofacial Orthop.

[32] Cho H-N, Yoon HJ, Park JH, Park Y-G, Kim S-J (2021). Effect of extraction treatment on upper airway dimensions in patients with bimaxillary skeletal protrusion relative to their vertical skeletal pattern. Korean J Orthod.

[33] Blackham SS. A study of short-term skeletal, dental, and soft tissue effects of class II malocclusions treated with Invisalign® with Mandibular Advancement feature or Twin Block appliance compared with historical controls. Text. 2020.

[34] Ehsani S, Nebbe B, Normando D, Lagravere MO, Flores-Mir C (2015). Short-term treatment effects produced by the twin-block appliance: a systematic review and meta-analysis. Eur J Orthod.

[35] Londono LCR. Maxillary and mandibular expansion treatment with Invisalign First: a retrospective study of virtual prediction Versus Clinical Outcomes. The University of Alabama at Birmingham; 2021.

[36] Clark W, Clark WJ. Twin block functional therapy. JP Medical Ltd; 2014.

[37] Coughlan A, Hennessy J, Najjar A, Auyang E, Batanghari W, Cartwright C. Invisalign: Orthodontics Unwired. Kellogg School of Management Cases 2017:1–18.

