# Supplementary material for: Comparison of invisalign mandibular advancement and twin-block on upper airway and hyoid bone position improvements for skeletal class II children: a retrospective study
Source: BMC Oral Health. 2023 Sep 13;23:661. doi: 10.1186/s12903-023-03295-2 (PMC10500932; doi:10.1186/s12903-023-03295-2)
Supplement: Supplementary file 1 — Additional file 1: The additional file 1 includes 4 figures depicting pre- and post-treatment data, reflecting two cases each from the MA group and the TB group: sFig 1, Case #1 of MA group. A-C, pre-treatment photos; D-F, post-treatment photos; G, pre-treatment 3D rendering of upper airway volume (Na-V = 2099.9 mm3, Or-V = 11799.5 mm3, Hy-V = 4059.9 mm3); H, post-treatment 3D rendering of upper airway volume (Na-V = 4532.5 mm3, Or-V = 13030.1 mm3, Hy-V = 5425.0 mm3); sFig 2, Case #2 of MA group. A-C, pre-treatment photos; D-F, post-treatment photos; G, pre-treatment 3D rendering of upper airway volume (Na-V = 8051.5 mm3, Or-V = 11968.1 mm3, Hy-V = 3609.2 mm3); H, post-treatment 3D rendering of upper airway volume (Na-V = 8706.8 mm3, Or-V = 13124.8 mm3, Hy-V = 4507.7 mm3); sFig 3, Case #1 of TB group. A-C, pre-treatment photos; D-F, post-treatment photos; G, pre-treatment 3D rendering of upper airway volume (Na-V = 1067.5 mm3, Or-V = 6957.5 mm3, Hy-V = 4452.4 mm3); H, post-treatment 3D rendering of upper airway volume (Na-V = 2257.2 mm3, Or-V = 8114.4 mm3, Hy-V = 5513.1 mm3); sFig 4, Case #2 of TB group. A-C, pre-treatment photos; D-F, post-treatment photos; G, pre-treatment 3D rendering of upper airway volume (Na-V = 5512.2 mm3, Or-V = 13793.8 mm3, Hy-V = 4186.8 mm3); H, post-treatment 3D rendering of upper airway volume(Na-V = 5002.4 mm3, Or-V = 21058.7 mm3, Hy-V = 5914.9 mm3). [file 12903_2023_3295_MOESM1_ESM.pdf]

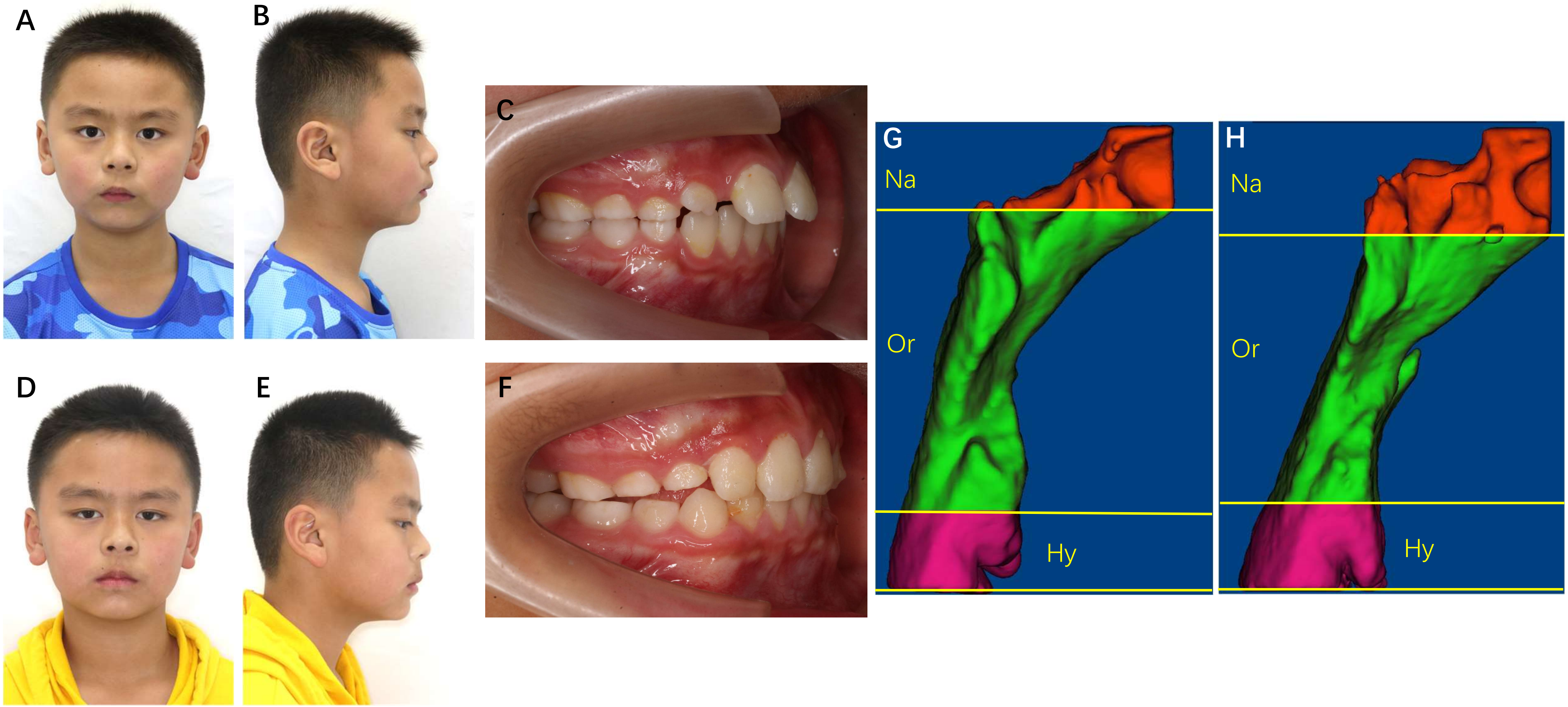

sFig 1. Case #1 of MA group. A-C, pre-treatment photos; D-F, post-treatment photos; G, pre-treatment 3D rendering of upper airway volume (Na-V = 2099.9 mm^3, Or-V = 11799.5 mm^3, Hy-V = 4059.9 mm^3); H, post-treatment 3D rendering of upper airway volume (Na-V = 4532.5 mm^3, Or-V = 13030.1 mm^3, Hy-V = 5425.0 mm^3).

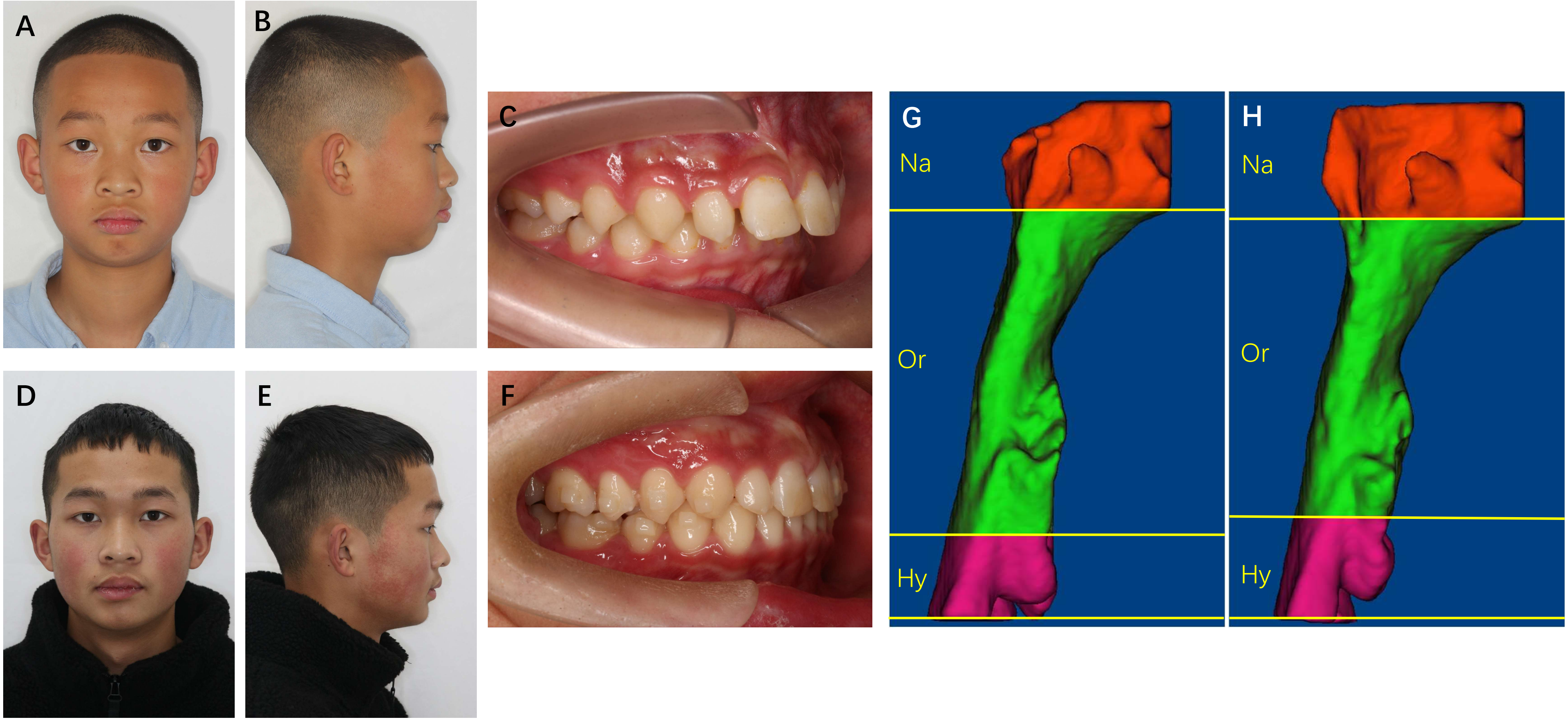

sFig 2. Case #2 of MA group. A-C, pre-treatment photos; D-F, post-treatment photos; G, pre-treatment 3D rendering of upper airway volume (Na-V = 8051.5 mm^3, Or-V = 11968.1 mm^3, Hy-V = 3609.2 mm^3); H, post-treatment 3D rendering of upper airway volume (Na-V = 8706.8 mm^3, Or-V = 13124.8 mm^3, Hy-V = 4507.7 mm^3).

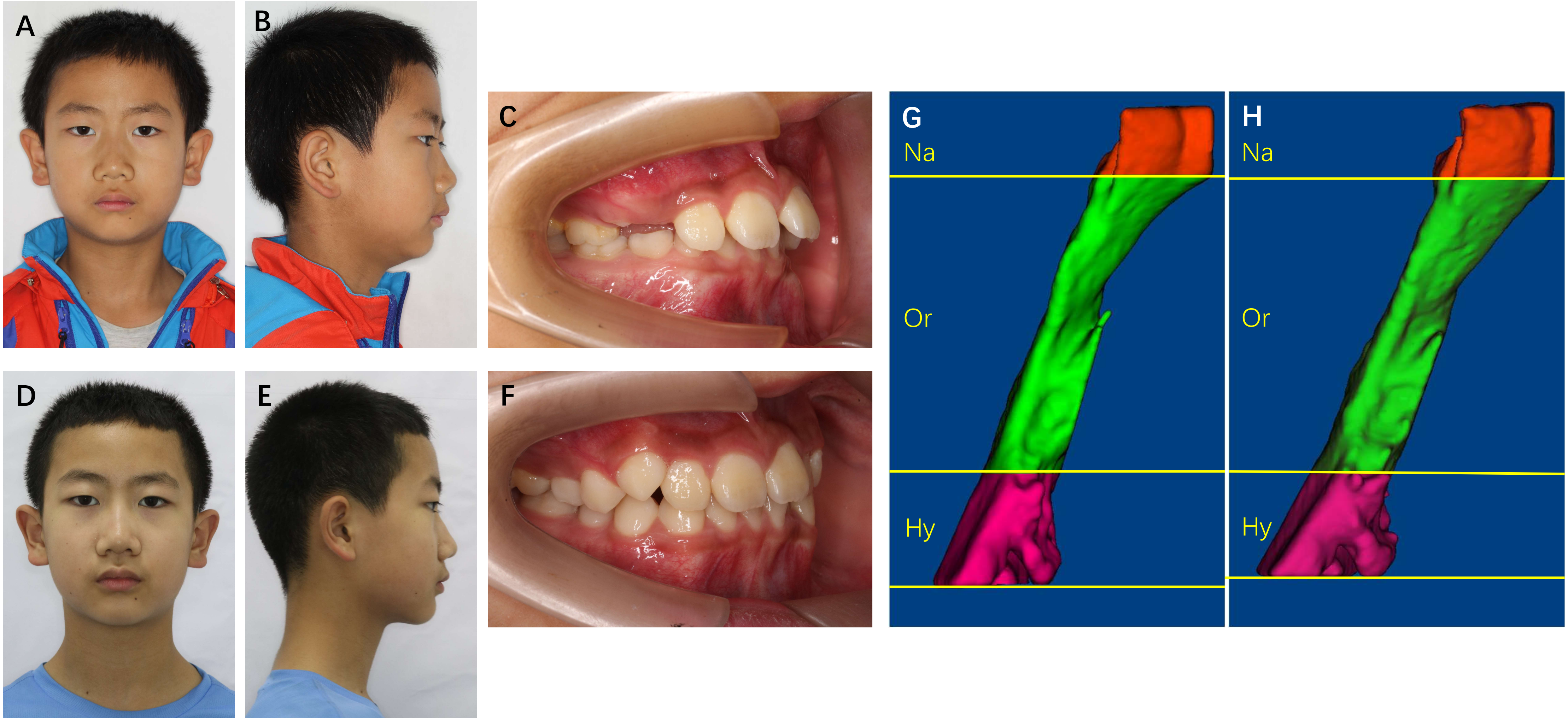

sFig 3. Case #1 of TB group. A-C, pre-treatment photos; D-F, post-treatment photos; G, pre-treatment 3D rendering of upper airway volume (Na-V = 1067.5 mm^3, Or-V = 6957.5 mm^3, Hy-V = 4452.4 mm^3); H, post-treatment 3D rendering of upper airway volume (Na-V = 2257.2 mm^3, Or-V = 8114.4 mm^3, Hy-V = 5513.1 mm^3).

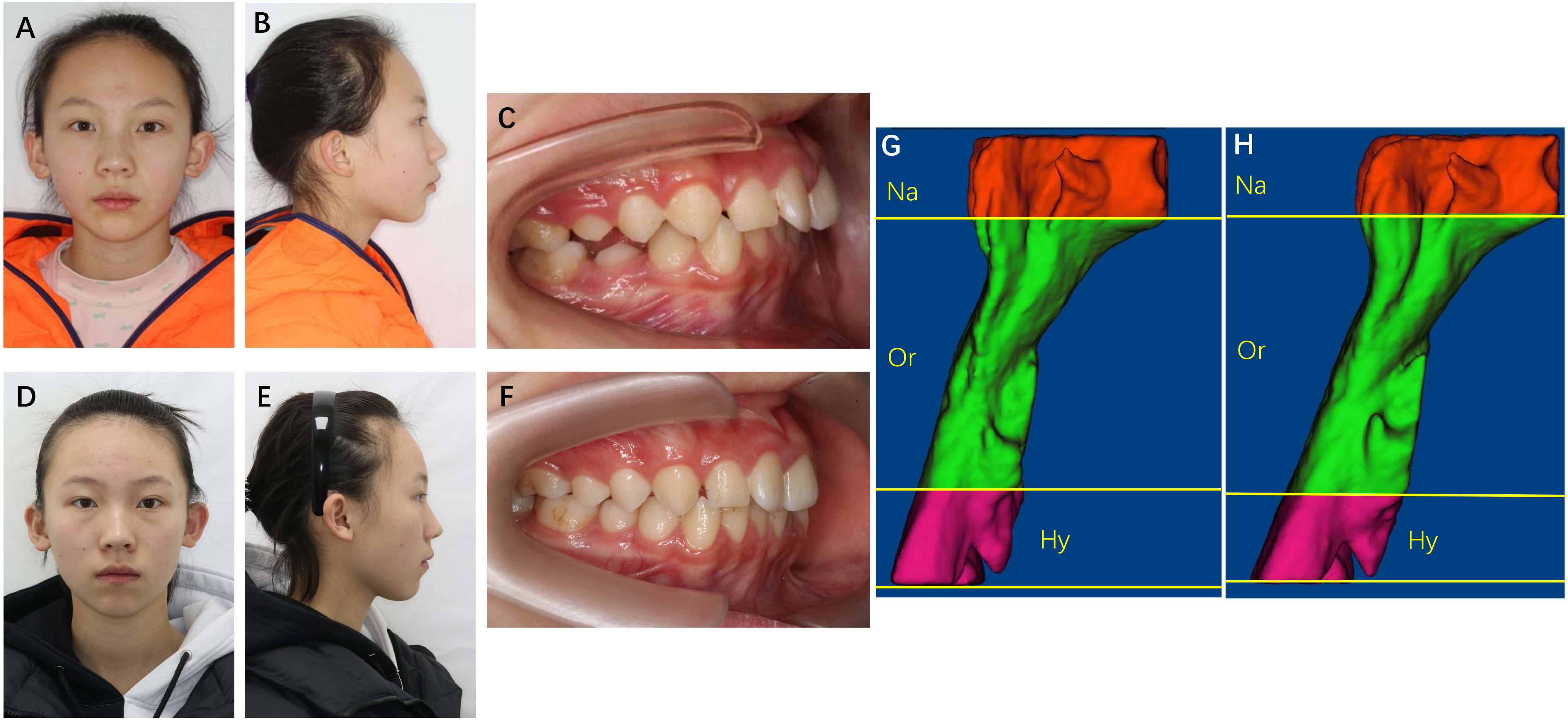

sFig 4. Case #2 of TB group. A-C, pre-treatment photos; D-F, post-treatment photos; G, pre-treatment 3D rendering of upper airway volume (Na-V = 5512.2 mm^3, Or-V = 13793.8 mm^3, Hy-V = 4186.8 mm^3); H, post-treatment 3D rendering of upper airway volume(Na-V = 5002.4 mm^3, Or-V = 21058.7 mm^3, Hy-V = 5914.9 mm^3).
